# Right Transcephalic Ventriculo-Subclavian Shunt in the Surgical Treatment of Hydrocephalus—An Original Procedure for Drainage of Cerebrospinal Fluid into the Venous System

**DOI:** 10.3390/jcm12154919

**Published:** 2023-07-26

**Authors:** Mircea Liţescu, Daniel Alin Cristian, Violeta Elena Coman, Anwar Erchid, Iancu Emil Pleşea, Anca Bordianu, Corina Veronica Lupaşcu-Ursulescu, Costin George Florea, Ionuţ Simion Coman, Valentin Titus Grigorean

**Affiliations:** 1 Discipline of Surgery and General Anesthesia—“Sf. Ioan” Clinical Emergency Hospital, 2nd Department, Faculty of Dental Medicine, “Carol Davila” University of Medicine and Pharmacy, 37 Dionisie Lupu Street, 020021 Bucharest, Romania; mircea.litescu@umfcd.ro; 2 General Surgery Department, “Sf. Ioan” Clinical Emergency Hospital, 13 Vitan-Bârzeşti Road, 042122 Bucharest, Romania; 3 Discipline of General Surgery—“Colţea” Clinical Hospital, 10th Department—General Surgery, Faculty of Medicine, “Carol Davila” University of Medicine and Pharmacy, 37 Dionisie Lupu Street, 020021 Bucharest, Romania; daniel.cristian@umfcd.ro; 4 General Surgery Department, “Colţea” Clinical Hospital, 1 Ion C. Brătianu Boulevard, 030167 Bucharest, Romania; 5 Discipline of General Surgery—“Bagdasar-Arseni” Clinical Emergency Hospital, 10th Department—General Surgery, Faculty of Medicine, “Carol Davila” University of Medicine and Pharmacy, 37 Dionisie Lupu Street, 020021 Bucharest, Romania; elena.coman@umfcd.ro (V.E.C.); valentin.grigorean@umfcd.ro (V.T.G.); 6 General Surgery Department, “Bagdasar-Arseni” Clinical Emergency Hospital, 12 Berceni Road, 041915 Bucharest, Romania; erchid.anwar@yahoo.com (A.E.); costinflorea1990@gmail.com (C.G.F.); 7 Pathology Department, “Bagdasar-Arseni” Clinical Emergency Hospital, 12 Berceni Road, 041915 Bucharest, Romania; pie1956@yahoo.com; 8 Discipline of Plastic and Reconstructive Surgery—“Bagdasar-Arseni” Clinical Emergency Hospital, 9th Department—Plastic and Reconstructive Surgery, Pediatric Surgery, Faculty of Medicine, “Carol Davila” University of Medicine and Pharmacy, 37 Dionisie Lupu Street, 020021 Bucharest, Romania; anca.bordianu@umfcd.ro; 9 Plastic Surgery and Reconstructive Microsurgery Department, “Bagdasar-Arseni” Clinical Emergency Hospital, 12 Berceni Road, 041915 Bucharest, Romania; 10 Discipline of Radiology and Medical Imagistics, 2nd Surgery Department, Faculty of Medicine, “Grigore T. Popa” University of Medicine and Pharmacy, 16 University Street, 700115 Iaşi, Romania; corina.ursulescu@gmail.com; 11 Radiology and Medical Imagistics Department, “Sf. Spiridon” County Emergency Hospital, 1 Independenţei Boulevard, 700111 Iaşi, Romania

**Keywords:** surgical education, new surgical procedure

## Abstract

The objectives of this article are to present an original surgical procedure for the temporary or definitive resolution of hydrocephalus, in the case of repeated failure of standard treatment techniques, and to present a case that was resolved using this surgical technique. Materials and methods: We present the case of a 20-year-old male patient with congenital hydrocephalus who underwent a number of 39 shunt revisions, given the repetitive dysfunctions of various techniques (ventriculo-peritoneal shunt, ventriculo-cardiac shunt). The patient was evaluated with the ventricular catheter externalized at the distal end and it was necessary to find an emergency surgical solution, considering the imminent risk of meningitis. The patient was also associated with the diagnosis of acute lithiasic cholecystitis. Results and discussions: The final chosen solution, right ventriculo-venous drainage using the cephalic vein, was a temporary surgical solution, but there are signs that this procedure can provide long-term ventricular drainage. Conclusions: Transcephalic ventriculo-subclavian drainage represents an alternative technical option, which can be used when established options become ineffective.

## 1. Introduction

Identifying the etiology of hydrocephalus, and the medical or surgical treatment of its cause, represent objectives that can only be achieved in a small number of cases [1,2]. The surgical treatment alternative is represented by ventricular drainage to structures or cavities that can take over variable amounts of cerebrospinal fluid (CSF) [3,4].

The technical variants adopted for the evacuation of excess CSF from the ventricular cavities are very numerous and have appeared as a necessity, given the imperfections of the reported methods [5,6]. Improving the results of these techniques has been a constant concern, given the inconveniences or complications specific to each method applied. Improving the quality of the tubing or pressure-modulating valves has improved postoperative outcomes [7,8,9,10].

The Imagined surgical techniques for the evacuation of excess CSF are diverse (ventriculo-peritoneal shunt, ventriculo-cardiac shunt, ventriculocisternostomy, ventriculostomy III). In general, these techniques are well tolerated and solve these patients’ problems for long periods [11,12,13,14,15,16,17].

The choice of the type of drainage takes into account a number of parameters: age, type of hydrocephalus, the severity of the condition, associated disorders, operative risks, possible inconveniences and complications, temporary or definitive nature of the chosen surgical solution, etc. [18,19].

Unfortunately, there are situations in which the chosen surgical procedure cannot ensure the long-term drainage of excess CSF, either due to malfunctions of the materials used (tubing, valves), due to the onset of complications in the structures that receive the excess CSF, or of complications at the level of cerebral structures (epi- and subdural hematomas, chronic subdural hygromas, septic complications, pneumoencephaly, post-shunt craniostenoses, etc.) [20,21,22,23,24,25,26,27,28].

Currently, the gold standard for the surgical solution of hydrocephalus is the ventriculo-peritoneal shunt. An efficient and well-tolerated procedure, the drainage of the peritoneal cavity of the CSF has spread widely and represents, in many cases, the first therapeutic option. Unfortunately, the range of complications specific to this technique is quite extensive: the obstruction or disconnection of the distal catheter, septic complications in the peritoneum or intraperitoneal organs, CSF pseudocyst, CSF ascites, bowel obstruction, inguinal hernia and hydrocele, visceral perforations, peritoneal metastases from central nervous system tumors, etc. [29,30,31,32,33,34,35,36]. Failure rates in ventriculo-peritoneal shunts have been estimated at percentages between 11–25% within the first year after initial shunt placement [37,38,39,40], with most references reporting a significantly higher number of shunt revisions among pediatric patients compared to adults [29,39,40].

Ventriculo-atrial shunts are frequently associated with septic or immunologically mediated complications (shunt nephritis), gas embolism, venous thrombosis, rhythm disorders, valvular lesions, interventricular perforation, intracranial hypotension, etc. [12,41,42,43,44,45,46,47,48,49,50,51]; rarely, lumbo-peritoneal shunts can generate arachnoiditis or radiculopathies [52,53].

The existence of such repetitive complications requires shunt revisions or choosing another surgical technique, with a progressive reduction of the chances of achieving long-term functionality. A series of technical variants already tried have proven limited efficiency, associated with severe complications, and used only in certain particular cases (ventriculo-pleural shunt, ventriculo-gallbladder shunt, ventriculo-ureteral shunt, lumbo-ureteral shunt, ventriculo-mastoid shunt, etc). [54,55,56,57,58].

## 2. Materials and Methods

We present the case of a 20-year-old male patient diagnosed with congenital hydrocephalus treated by a surgical procedure for the first time six months after birth (ventriculo-peritoneal shunt). During the next four years, the functionality of the drainage was good but, later, a CSF pseudocyst occurred. This complication led to surgical evacuation of the pseudocyst and repositioning of the distal catheter. Such episodes arrived at various time intervals, constraining the surgical team to change the type of placement to a biventriculo-atrial shunt with a low-pressure valve at the age of 17. Unfortunately, 13 months later, the patient presented with headache, vomiting and unsystematized static and dynamic balance disorders. The cerebral CT scan performed at admission revealed enlarged cerebral ventricles. Due to this situation, a new surgery followed in order to convert the drainage to a unishunt biventriculo-peritoneal system.

In the next three years, new surgical procedures were interspersed in 12 situations externalizing the shunt for several days, the time needed to rest the peritoneum, performing investigations on the quality of the CSF and establishing the therapeutic strategy to follow (subsequently). Thus, about 38 ventriculo-peritoneal shunt revisions and a ventriculo-cardiac shunt took place in a relatively short interval.

**Figure 1:**
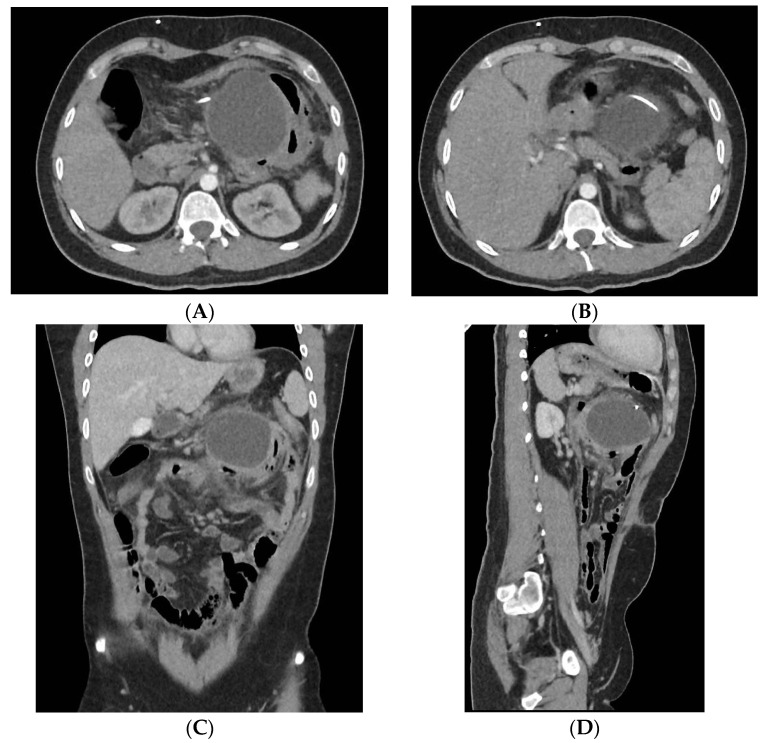
Abdominal Computer Tomography (CT) scan sections reveal a CSF pseudocyst—a well-defined liquid collection, round in shape, with axial dimensions of approximately 77 mm. Axial planes (**A**,**B**), coronal plane (**C**), and sagittal plane (**D**) of the CT scan.

The recurrence of the intracranial hypertension symptoms after this long succession of surgeries brought the patient, once again, to the emergency department. Upon hospital admission, the imaging exams showed the occurrence of a new CSF pseudocyst (Figure 1A–D) and acute lithiasic cholecystitis. The neurosurgical team externalized the distal end of the ventricular drainage catheter from the peritoneum as an emergency procedure.

The surgical evaluation confirmed the existence of the two pathological entities that required surgical resolution. Although the rate of success after the evacuation of the pseudocyst and repositioning of the intraperitoneal catheter was relatively low, this was attempted considering the laparoscopic approach required to solve the acute lithiasic cholecystitis. On this occasion, we observed an intense process of bowel adhesions (predominantly in the inframesocolic space) and we performed adhesiolysis, retrograde laparoscopic cholecystectomy, pseudocyst evacuation and the repositioning of the distal tip of the catheter in the lower abdomen, after reconnection to the drainage system. The subhepatic drainage tube was removed 24 h postoperatively.

**Figure 2:**
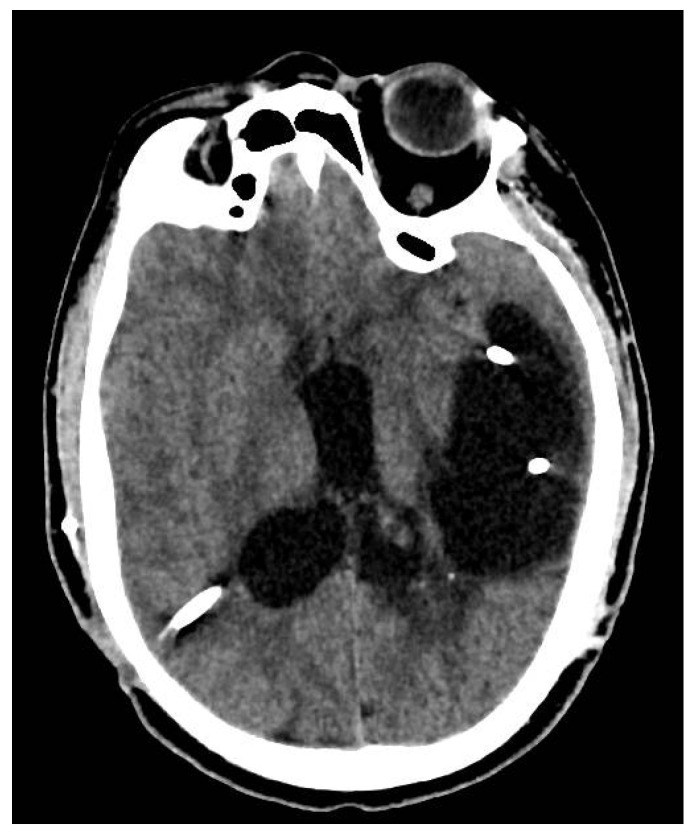
Cerebral CT scan—left temporal cystic cavity of 77/48 mm; similar infratentorial image of 29/19 mm in the axial plane, adjacent to the pons; ventricular drain tubes at the level of the body of left lateral ventricle, body and posterior horn of the right lateral ventricle.

**Figure 3:**
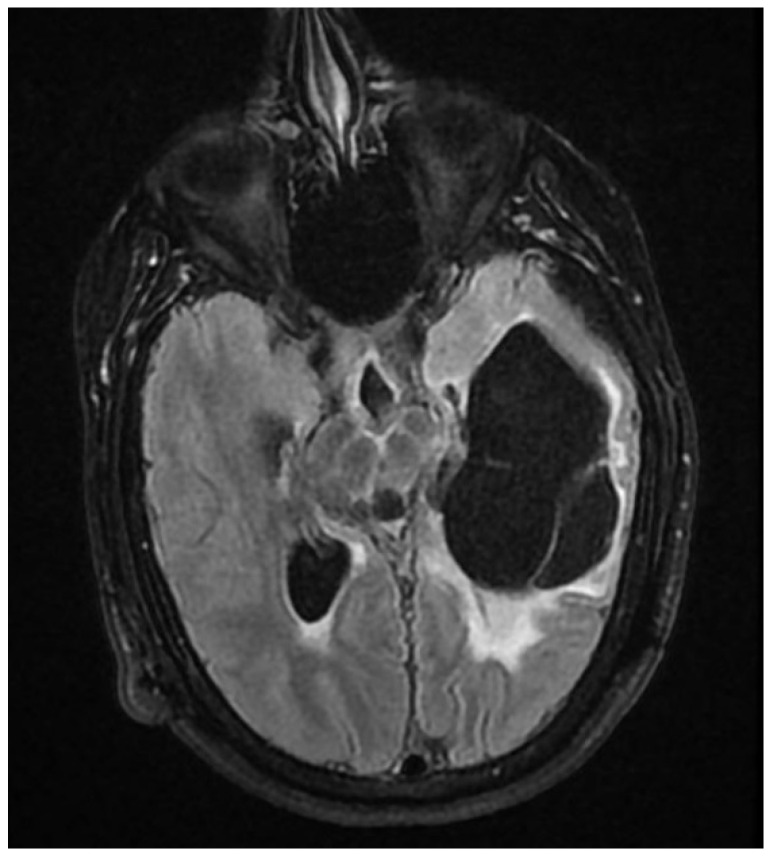
Ax Flair T2 head MRI—septate cystic cavity, with identical signal to CSF, maximum dimensions of 80.3/33/47.1 mm, located temporo-occipital on the left, with neighboring glial changes and presence of the intracavitary tube.

The immediate postoperative evolution was favorable, both surgically and neurologically for approximately 14 days, until the intracranial hypertension phenomena reappeared, the patient presenting headache, vomiting, fever, altered state of consciousness, drowsiness and divergent strabismus. A cerebral CT scan and Magnetic Resonance Imaging (MRI) showed active hydrocephalus (Figure 2 and Figure 3), which again required the externalization of the distal end of the ventricular catheter.

**Figure 4:**
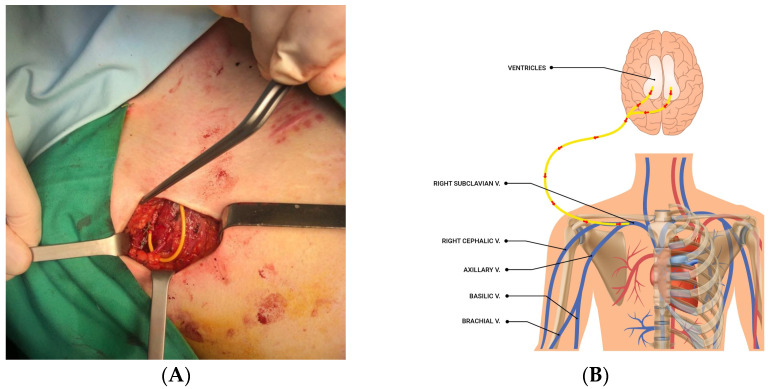
Intraoperative aspect with the catheterization of the right cephalic vein (**A**) and a schematic diagram of the drainage (**B**).

**Figure 5:**
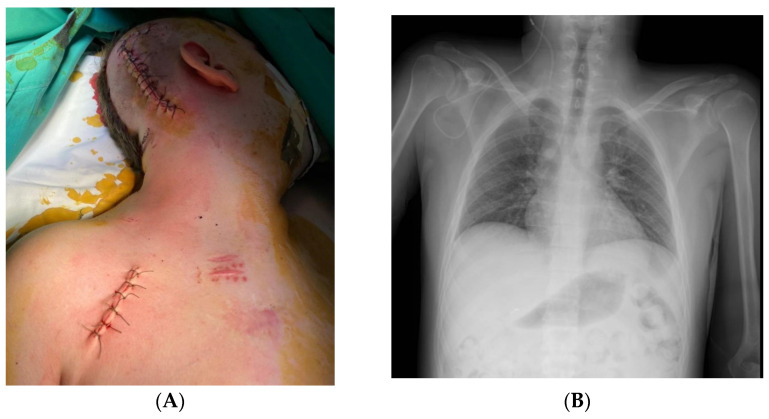
Postoperative clinical aspect (**A**) and postoperative X-ray aspect of the right ventriculo-subclavian shunt (**B**).

**Figure 6:**
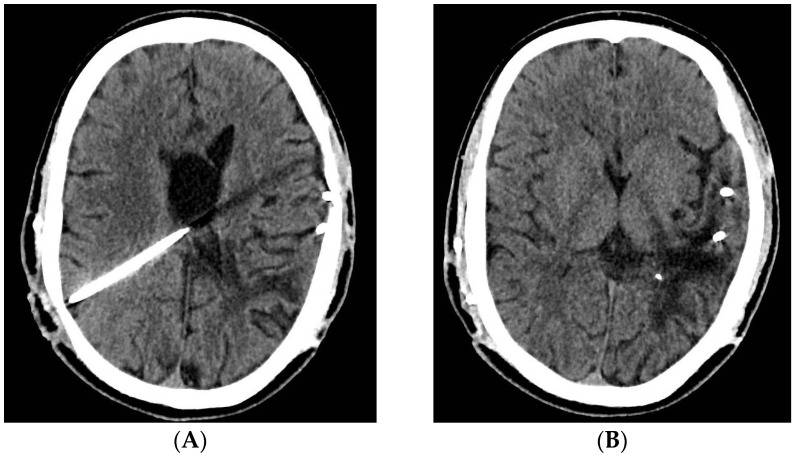
The postoperative aspect of cerebral CT scan—compared to the previous examinations, favorable evolution, with efficient ventricular drainage (**A**) and dimensional decrease of the ventricular system and the left temporal cystic lesion, indistinguishable from the adjacent left ventricle (**B**).

Faced with this therapeutic impasse, we found the solution of catheterizing the right cephalic vein, a tributary of the superior cava system, as the equivalent of the ventriculo-cardiac shunt. The vein was identified in the right deltopectoral space and catheterized with the distal end of the ventricular tubing (Figure 4A,B and Figure 5A,B), with CSF flow modulated by the right retroauricular valve. The distal end (intraperitoneal) of the drainage duct was initially abandoned in the previous position, then suppressed. The clinical and imaging (Figure 6A,B) outcome was favorable four months after the surgery.

## 3. Results

The evacuation of excess CSF from the ventricular system represents an objective that can be solved by medical or surgical means, to avoid severe intracranial hypertension, with consequences on the cerebral noble substance. Due to the low efficiency of the medication, it is used solely in mild forms and as a preoperative preparation [59].

The surgical treatment uses three leading solutions:

− endoscopic internal drainage (ventriculostomy III with/without aqueductoplasty, perforation of the supraoptic blade, posterior ventriculostomy).

The main beneficiaries of these procedures are patients with obstructive hydrocephalus by posterior cerebral fossa tumors, those with CSF circulation disorders (Sylvius aqueduct stenosis, Dandy–Walker malformations) and skull base malformations. The multiple advantages of these surgical procedures are overshadowed by: the limitation of applicability to the aforementioned pathologic conditions, bleeding from the cervical plexus, risk of injury to the basal artery branches, closure of the surgically created communication or subdural hematoma [16,60,61,62,63,64,65,66,67,68].

− external drainage of the CSF and its collection in an external reservoir is acceptable as a temporary solution in the case of association with meningitis or intraventricular bleeding [69,70,71].
− extracranial drainage of the CSF is the most extensive method and it is applied to all patients, with the choice of the technical solution depending on several criteria: age, generating cause, associated disorders, operating risks, temporary or definitive nature of the procedure, etc. [4,11,12,18,72,73].

The efficiency of drainage in the cephalic vein (which can be extended to the superior vena cava and right heart, respectively) with results and possible complications similar to ventriculo-cardiac drainage, but using another approach, confirms the validity of the procedure and gives hope for the alternative surgical treatment of hydrocephalus in case of the appearance of complications related to established surgical procedures.

In the period elapsed from the moment our surgical team applied this imagined therapeutic solution, no complications related to the neurological condition and the operative act were found.

## 4. Discussion

Although physiological considerations suggest that the drainage of excess CSF from active hydrocephalus should drain into a venous segment, the well-established surgical method is represented by the ventriculo-peritoneal shunt. The peritoneum provides a generous resorption surface, which tolerated the CSF well. The resorptive function of the peritoneum can be canceled or reversed by certain factors, which are partially known: immunological, septic, mechanical, allergic factors, etc. Under these conditions, the ventriculo-peritoneal shunt becomes non-functional due to the appearance of an intraperitoneal complication: CSF pseudocyst, CSF ascites, plastic peritonitis, etc. Thus, a ventriculo-peritoneal shunt, well tolerated for long periods of time, can become quite suddenly ineffective. Reinterventions that propose shunt revision, with the possible solving of intraperitoneal surgical complications, represent the solution, but with the diminished possibility of finding a long-term resolution using the same surgical procedure [29,33,74,75,76,77,78].

This problem determines the quest for another surgical solution (endoscopic internal drainage or ventriculo-cardiac shunt). The alternative methods mentioned above resolve the situation, but they are also burdened by certain complications as previously noted. Thus, a therapeutic impasse that can endanger the neurological condition and even the patient’s life can appear in many cases. Finding alternative solutions has been a permanent concern, but unfortunately no superior treatment options have been identified.

Analyzing the particular situation of our patient, considering that the iterative repositioning of the catheter is doomed to failure, that the indication of intraperitoneal drainage was not an option and that the transjugular path (ventriculo-cardiac shunt) was exhausted, we identified the solution of placing the ventricular catheter in the right cephalic vein, thus accessing the superior cava system, with the possibility of progression up to the level of the right side of the heart.

The reasons considered In choosing this surgical approach were: the sufficient pressure gradient between the cerebral ventricular system (even more so in conditions of hydrocephalus) and central venous pressure, the convenience of access (the catheter crosses a short subcutaneous segment to the deltopectoral space, where the surgeon will find the right cephalic vein), reduced surgical risks, physiological considerations (the procedure is an equivalent of a ventriculo-cardiac shunt using another access route), the ease of re-accessing the valve and the venous approach (if necessary), the excellent tolerance of CSF in the venous system, etc.

The pressure gradient ensures adequate drainage (under 20 mmHg in the ventricular system in the absence of hydrocephalus [79] and 2–6 mmHg in the upper cava system [80]). The much higher values in hydrocephalus cause an unhindered discharge of excess CSF (but modulated by the pressure valve) into a venous bloodstream, which can take up any amount.

The catheter inserted into the cephalic vein can be advanced to the level of the large veins tributary to the superior vena cava, superior cavities, or up to the level of the right heart, thus obtaining a classic ventricular shunt equivalent. In our case, the catheter was placed in the right subclavian vein, using the right transcephalic route.

Some pathologic circumstances may limit the use of this access route to the superior vena cava system, such as the use of cephalic vein in a previous history for angioaccess procedures, thrombosis of superficial veins of the right upper limb, tumoral disorders, keloid scars of the deltopectoral space, orthopedic disorders of the shoulder, etc. In this case, the contralateral cephalic vein can be used, although the drainage tubing route is longer, with more risk of torsion and drainage malfunction.

Another circumstance that may contraindicate this procedure is the occurrence or persistence of complications related to the ventriculo-cardiac shunt previously practiced by the transjugular route (this complication was mentioned above). It is assumed that the drainage of excess CSF by the transcephalic/transsubclavian pathway reactivates or worsens a previously observed pathological condition (e.g., shunt nephritis).

In the event of a dysfunction of this type of shunt, the revision takes little effort to perform, the access being convenient, on the ventricular catheter, valve, or distal segment of the drainage tubing.

In the literature, the ventriculo-subclavian shunt was described by a series of authors. Matsuoka et al., in 1993, published two case reports of a 64-year-old male and of a 65-year-old male, respectively, both of whom had their subclavian vein punctured through the infraclavicular approach, with positive results [81]. Another case reported by Evangelos et al. in 2017 presented a 4-year-old child with multiple ventriculo-peritoneal shunt revision surgeries and ventriculo-atrial failure due to distal catheter malfunction that was treated with the percutaneous placement of the peripheral catheter in the subclavian vein [82].

Using the right cephalic vein as the anatomic area of insertion of the ventricular shunt into the venous system is the innovative step of our procedure. The proposed surgical procedure did not raise any particular technical problems, and the intervention was carried out without complications. We did not study the flow of the cephalic vein preoperatively (Doppler ultrasound, Computer Tomography, phlebography), but the lack of other therapeutic options led us to use the cephalic vein as an access path to reach the subclavian vein whose venous flow we considered sufficient to absorb the excess CSF; the favorable clinical and imagistic evolution of the patient showed that this assumption was correct.

The test of time will prove whether the proposed method will be imposed as a therapeutic alternative to the well-established techniques, in the event of a therapeutic impasse, or as a first-option solution.

## 5. Conclusions

1. The ventriculo-subclavian shunt is an easy surgical procedure.
2. It is a solution for cases where the variants of standard surgical treatment have been exhausted.
3. It is a drainage solution of excess CSF in the superior vena cava system (equivalent to the established ventriculo-cardiac shunt).
4. It uses an access path that does not have anatomical/functional disadvantages.
5. Depending on the patency of the method and possible late complications, it can become a variant of a first-option treatment.
